# Efficacy and Safety of a Combined Aerobic, Strength and Flexibility Exercise Training Program in Patients with Implantable Cardiac Devices

**DOI:** 10.3390/jcdd9060182

**Published:** 2022-06-06

**Authors:** Maria Rosaria Squeo, Barbara Di Giacinto, Marco Alfonso Perrone, Massimo Santini, Maria Luisa Sette, Emanuele Fabrizi, Antonia Vaquer, Attilio Parisi, Antonio Spataro, Alessandro Biffi

**Affiliations:** 1 Institute of Sports Medicine, Sport e Salute, 00197 Rome, Italy; marysqueo@yahoo.it (M.R.S.); ext_barbrara.digiacinto@sportesalute.eu (B.D.G.); m.santini@rmnet.it (M.S.); marialuisa.sette@gmail.com (M.L.S.); e.fabrizi1@gmail.com (E.F.); antonio.spataro@coni.it (A.S.); a.biffi@libero.it (A.B.); 2 Department of Cardiology and University Sports Centre, University of Rome Tor Vergata, 00133 Rome, Italy; 3 Department of Cardiology, Fundacio Clinic, 08036 Barcelona, Spain; a.vaquersegui@gmail.com; 4 Department of Movement, Human and Health Science, University of Rome Foro Italico, 00135 Rome, Italy; attilio.parisi@uniroma4.it

**Keywords:** exercise training, cardiac rehabilitation, implantable cardiac devices, arrhythmias

## Abstract

**Purpose**: The “FIDE Project” (Fitness Implantable DEvice) was organized by the Institute of Sports Medicine and Science and the World Society of Arrhythmias with the aim of demonstrating the usefulness of exercise training in improving functional capacity in patients with implantable cardiac devices. **Materials and Methods**: Thirty sedentary patients were selected for the project (25 males and 5 females), with a mean age of 73 ± 5 years (range 44–94 years). Twenty-five were implanted with a Pacemaker (PM) and five with an Implantable Cardioverter Defibrillator (ICD). Atrial fibrillation/atrial flutter was present in ten (34%) patients, post-ischemic dilated cardiomyopathy in five (17.2%), sick sinus syndrome in six (20,7%), complete atrium-ventricular block in six (20.7%), hypertrophic cardiomyopathy in one (3.4%) and recurrent syncope in one (3.4%). The baseline assessment comprised cardiovascular examination, resting and stress ECG, cardiopulmonary exercise testing (V ˙O2peak), strength assessment of different muscle groups, and a flexibility test. The same measurements were repeated after 15–20 consecutive training sessions, over a 2-month period. The exercise prescription was set to 70–80% of HRR (Heart rate reserve) and to 50–70% of 1RM (1-repetition maximum, muscular force). The training protocol consisted of two training sessions per week performed in our institute, 90 min for each (warm-up, aerobic phase, strength phase and stretching) and one or more at home autonomously. **Results**: The cardiopulmonary testing after the training period documents a significant improvement in V ˙O2peak (15 ± 4 mL/kg/min vs. 17 ± 4; *p* = 0.001) and in work load (87 ± 30 watts vs. 108 ± 37; *p* = 0.001). Additionally, strength capacity significantly increased after the cardiac rehabilitation program, (quadriceps: 21 ± 18 kg vs. 29 ± 16 kg, *p* = 0.00003). Flexibility tests show a positive trend, but without statistical significance (sit-and-reach test: −19 ± 11 cm vs. −15 ± 11.7 cm; back-scratch test: −19 ± 11.6 cm vs. −15 ± 10 cm; lateral flexibility right −44 ± 1.4 cm vs. −43 ± 9.5 cm; left −43 ± 5 vs. −45 ± 8.7 cm). **Conclusion**: A brief period of combined aerobic, strength and flexibility exercise training (FIDE project) proved to be effective and safe in improving functional capacity in patients with cardiac implantable devices.

## 1. Introduction

The number of pacemakers (PM) and implantable cardioverter defibrillators (ICD) implanted in Italy is constantly increasing, with more than 25,000 devices inserted every year (426 per million) [1]. This increase is related to the reduced size of the devices, the increased physiological control of the cardiac electrical activity (also during exercise), and the results of follow-up studies on large cohorts [1].

Cardiac rehabilitation has been recognized as the most cost-effective intervention in ensuring favourable outcomes across a wide spectrum of cardiovascular diseases, reducing cardiovascular mortality, morbidity and disability, and increasing quality of life [2,3].

However, most of the studies are focused on patients with coronary artery disease, heart failure, post-cardiac surgery, or featuring cardiovascular risk factors [3]. In particular, several studies have shown that, in addition to being effective, cardiac rehabilitation programs including elements of supervised exercise are also safe for these cardiac patients, with an extremely low rate of adverse events and/or hospitalization [4,5].

Recent studies and consensus papers have demonstrated the efficacy and safety of cardiac rehabilitation even in patients with implantable cardiac devices [6,7,8]. ICDs are used to treat cardiac arrest by delivering shocks to terminate lethal cardiac arrhythmias. It is common after getting an ICD for patients to be afraid to exercise out of fear of receiving an ICD shock with elevated heart rates associated with exercise [9]. Additionally, clinicians are not often confident in prescribing exercise regarding intensity, duration, or frequency for improving aerobic capacity while not causing an ICD shock [9]. On the other hand, in patients with PMs, the device must provide heart rate acceleration during exercise, while keeping synchrony between atrial and ventricular contractions, to preserve optimal ventricle filling, in addition to detecting any arrhythmias [10].

Therefore, in the past, patients with implantable cardiac devices were often associated with a poor quality of life and a limitation of daily activities, including physical activity, which resulted in reduced exercise tolerance and functional autonomy [9,10].

In recent years, post-implantation rehabilitation programs have demonstrated clinical efficacy in terms of allowing patients to achieve improved functional capacity, reduced morbidity and improved quality of life through personalized and supervised training protocols [6,7,8]. In particular, several studies have demonstrated the efficacy and safety of rehabilitation programs with aerobic training in patients with ICD, without an increased risk of shocks [9,10,11,12].

In contrast, less data are available on the effects of a combined rehabilitation program (combined aerobic, strength and flexibility exercise training) in patients with PMs or ICDs.

Considering that cardiac rehabilitation with a combined aerobic program, including strength and flexibility training, has been largely shown to be effective in improving functional capacity and cardiovascular performance in patients with coronary artery disease [3,12,13], it is clinically relevant to consider whether such a combined program of cardiac rehabilitation is effective (and safe) even in patients with PMs or ICDs.

Therefore, the purpose of this study is to determine the efficacy and safety of a regular, monitored combined aerobic, strength and flexibility exercise training program in patients with an implantable device.

## 2. Materials and Methods

### 2.1. Study Group

Starting in 2014, the Institute of Sports Medicine and Science of the Italian Olympic Committee (CONI) decided to widen its field of interest also to the general population, not just elite athletes, but also subjects with cardiovascular risk factors or overt disease that can benefit from a tailored and supervised physical activity program, as a means of primary and secondary prevention. From a group of 93 patients undergoing our cardiac rehabilitation program in 2014, 30 patients with implantable cardiac devices that met the inclusion/exclusion criteria of the study protocol were selected.

These inclusion criteria were: at least 3 months since device implantation (PM or ICD), no orthopaedic issues that prevent exercise training, NYHA class I–II. The exclusion criteria were: third degree obesity, haemodynamic instability, recent infections.

The study was approved by the Institutional Review Board of the Institute of Sports Medicine (protocol code 8746/14) and all subjects signed an informed consent. The study was conducted in accordance with the Declaration of Helsinki. All patients were also under pharmacological therapy, as shown in Table 1.

### 2.2. Cardiovascular Rehabilitation Program

The cardiac rehabilitation program was divided into 3 phases:

*Phase 1: Clinical and Functional Capacity Evaluation*

Clinical evaluation of the 30 patients with implantable cardiac devices included medical history, physical examination, resting 12-lead electrocardiogram (ECG) and basal blood pressure measurement. The following functional capacity parameters were measured before and after the 8-week training program:

-Aerobic fitness test: Each patient underwent incremental, continuous, multistage cardiopulmonary exercise testing with a cycle ergometer. A graded exercise protocol, with increments of 25 watts (for females) or 30 watts (for males) every two minutes, maintaining rotation speed of 60–70 revolutions per minute, was adopted up to exhaustion or appearance of symptoms or electrocardiographic signs (arrhythmias, significant ST-segment abnormalities) or when blood pressure exceeded 200 mmHg systolic and/or 100 mmHg diastolic. The test was monitored using a breath by-breath gas analyser (Quark CPET, COSMED, Rome, Italy) measuring oxygen uptake, oxygen pulse and heart rate. V ˙O2 at lactate threshold was measured from the ratio of VE/VCO2 over VE/VO2 according to Binder et al. [14].

-Muscular strength tests: Muscular strength was estimated for upper limbs (biceps, triceps and deltoid) and lower limbs (quadriceps) with a 1-repetition maximum (1RM) according to Brzycki’s formula [15]. This formula uses a submaximal load and number of repetitions performed to indirectly estimate maximum strength, thus avoiding excessive pressure loads on the patient. The patient performed the various tests after an aerobic warm-up of 15 min on the cycle ergometer (50–55% of HRR) and between one test and the following there were 5 min of recovery. The limb measured was the dominant one, both for the upper and lower limbs.

-Flexibility tests: This evaluation was performed by adopting the sit-and-reach test, back-stretch test and lateral-mobility-of-the-trunk test. The sit-and-reach test aims at measuring the flexibility of the lower back and hamstrings. The subject leans forward slowly, without momentum movements, bringing the fingertips as far as possible while keeping the legs outstretched. The subject has to maintain the maximum elongation position for 2 s while the tester reads the result. The test is repeated twice and the best result is the one considered [16]. The objective of the back-stretch test is to evaluate the flexibility of the upper part of the body (shoulders). Placing one hand behind the head and back over the shoulder, subjects reach as far as possible down the middle of the back, with the palm touching the body and the fingers directed downwards. The other arm is placed behind the back, palm facing outward and fingers upward, and subjects reach up as far as possible while attempting to touch or overlap the middle fingers of both hands. The distance left between fingers or the distance they overlap is measured [17]. The lateral-mobility test is used to determine the degree of lateral flexibility of the spine. The individual flexes the torso laterally in the frontal plane. The tester detects the difference in centimetres between the starting position of the hand and the ending position in the maximum inflection point. The test was repeated for the contralateral side [18].

*Phase 2: Training Program*

**Figure 1:**
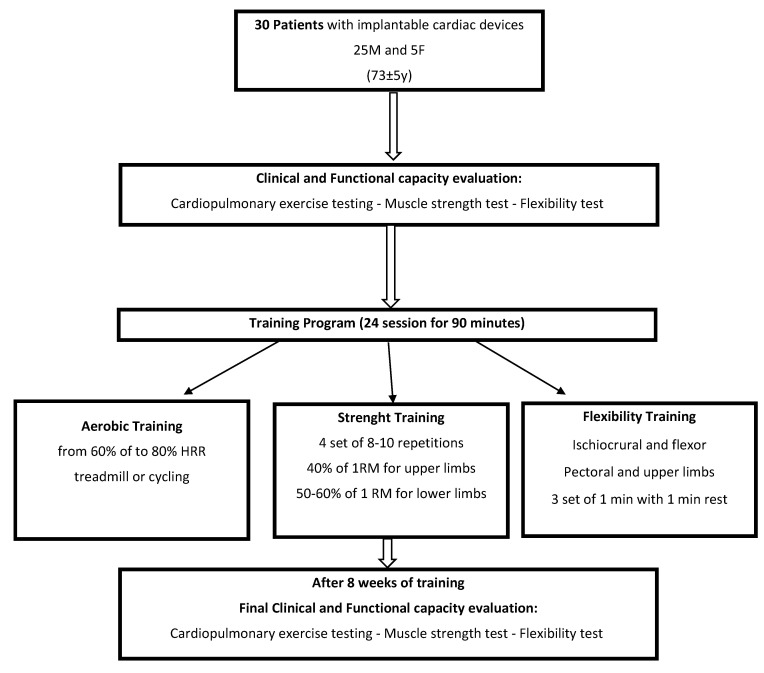
Training program of the patients studied.

The training program consisted of 3 training sessions per week (2 in an institute and 1 at home), for a total of 24 sessions. Each session lasted for about 90 min and comprised aerobic, strength and flexibility training. Training sessions were electrocardiographically monitored with telemetry (Mortara, Casalecchio di Reno, Italy) and the blood pressure was measured at rest at the beginning and at the end of each training session. Concerning the aerobic part, patients were assigned with an initial target heart rate of 60% of the heart rate reserve (HRR) that was then progressively titrated to 80% of HRR, with absolute upper exercise limit of 10 beats below the ICD activation threshold for the aerobic part of the training session (40 min each session). Moderate to Vigorous Continuous Aerobic Training consisted of walking/running on the treadmill or cycling on the cycle ergometer with continuous ECG monitoring. Strength training was based on the patient’s 1-RM following the Brizscky formula and consisted of 4 sets of 8–10 repetitions with one minute of rest between each set of: one-arm lateral rises, one-arm bicep curl, one-arm triceps extensions per muscle group performed with dumbbell, leg press, and leg extensions. Strength training started from approximately 40% of 1-RM for upper limb exercises and 50–60% 1-RM for lower limbs, in order to ensure correct execution of each exercise and thus preventing pressure overload. Flexibility training was performed at the end of each training session, and consisted of ischiocrural and flexor stretching for 1 min using the Flexability posterior machine, pectoral and upper limbs stretching for 1 min in Doorway Pectoral Stretch position, and quadriceps stretching for 1 min in one-leg flex standing position. Each flexibility exercise was performed three times with 1 min rest between sets. Respiration and relaxation exercises were also performed at the end of the flexibility session for about 5 min to finish the cool-down (Figure 1).

*Phase 3: Assessment and Outcomes*

The initial tests were repeated after 8 weeks of cardiovascular rehabilitation program to evaluate whether differences did occur in comparison with pre-training status.

### 2.3. Statistical Analysis

Data are expressed as mean ± SD. The differences between pre- and post-exercise training were evaluated by paired *t*-test. A *p* value ≤ 0.05 was considered statistically significant. The statistical analyses were performed using the commercial statistical software package Medcalc (Version 18.2.1, Ostend, Belgium).

## 3. Results

All patients successfully completed the study protocol and there were no dropouts. All clinical data assembled from patients were maintained in an institutional database. The clinical and demographic characteristics of the 30 patients are shown in Table 1. Patients were 73 ± 5 years old (range 44–94 years), 25 males (83%) and 5 females, twenty-five with a PM and five with an ICD. Atrial fibrillation/atrial flutter was diagnosed in 37% (*n* = 11) of patients, post-ischemic dilated cardiomyopathy in 17.2% (*n* = 5), sick sinus syndrome in 20.7% (*n* = 6), complete atrioventricular block in 20.7% (*n* = 6), hypertrophic cardiomyopathy in 3.4% (*n* = 1) and recurrent syncope in 3.4% (*n* = 1). Sixteen patients had a dual chamber cardiac pacing device for sick sinus syndrome or complete atrioventricular block, nine patients were implanted with a single chamber cardiac pacing device for atrial fibrillation control, five patients had an ICD for mixed forms of dilated cardiomyopathy, post-ischemic dilated cardiomyopathy and hypertrophic cardiomyopathy. With respect to cardiovascular (CV) risk factors, hypertension was the most frequent risk factor (30%), followed by dyslipidemia (20%), obesity (17%), diabetes mellitus (13%) and smoking (10%).

The differences in measured variables between pre- and post-training are shown in Table 2.

V ˙O2peak significantly increased from 15 ± 3.8 mL/kg/min to 17 ± 4 (*p* = 0.001) after the training program. Furthermore, work load significantly improved from 87 ± 30 watts at baseline to 108 ± 37 watts after cardiac rehabilitation (*p* = 0.001). V ˙O2 at lactate threshold (LT V ˙O2) shows a positive trend increasing from 10 ± 2.2 to 11 ± 2.2 mL/kg/min. The post-training evaluation shows a significant reduction of diastolic blood pressure values (from 77 ± 10.7 mmHg to 72 ± 11.1 mmHg; *p* = 0.009) and a significant increase in heart rate response to exercise (from 96 ± 19.5 bpm to 106 ± 22.6; *p* = 0.008). No significant change occurred in oxygen pulse.

Muscle strength significantly increased following the cardiac rehabilitation program in all main muscles evaluated (Table 2). The muscle strength of quadriceps increased from 21 ± 18 kg to 29 ± 16 (*p* = 0.000003), the muscle strength of biceps brachii increased from 11 ± 5 to 15 ± 9 kg (*p* = 0.02), the strength of triceps brachii increased from 7 ± 3 to 9 ± 4 kg (*p* = 0.003) and the muscle strength of deltoid increased from 6 ± 2 to 7 ± 3 kg (*p* = 0.01).

Regarding flexibility measurements, the sit-and-reach test showed a significant improvement after training (−19 ± 11 cm vs. −15 ± 11 cm *p =* 0.003), while the other parameters (back-scratch test right: −19 ± 11.6 cm vs. −15 ± 10 cm; back-scratch test left −21 ± 17.7 cm vs. −18 ± 13.5 cm; lateral flexibility right: −44 ± 1.4 cm vs. −43 ± 9.5 cm; left −43 ± 6 vs. −45 ± 8.7 cm) showed a positive trend (Table 2). No patient experienced adverse cardiac events and there were no ICD shocks

## 4. Discussion

The main findings of the present study are that combined aerobic, strength and flexibility exercise training in patients with implanted cardiac devices is effective in improving functional capacity and muscular strength without safety concerns.

Previous studies [2,3,4,5,6,7] had already demonstrated the positive effects and safety of aerobic training program alone in patients with implantable cardiac devices. In the HF-ACTION study [19], patients with heart failure and implanted cardiac devices underwent aerobic exercise training and showed no evidence of increased ICD shocks. It was concluded that exercise should not be prohibited in patients with implanted cardiac devices [19]. Pandey et al. [20] also reported that, among patients with heart failure and implanted cardiac devices, aerobic exercise training was associated with significant improvements in cardiorespiratory fitness (CRF) and a low likelihood of ICD shocks.

Cardiac rehabilitation programmes produce positive effects through the modification of cardiovascular disease risk factors and by promoting gains in CRF [21,22,23]. Higher CRF is associated with a reduced risk of cardiovascular mortality and morbidity, as is the magnitude of gains in fitness due to exercise training [21,22,23,24,25].

Our study confirms these results [19,20] but also extend the efficacy and safety of exercise training to a more comprehensive training program that also includes strength and flexibility exercises.

Indeed, eight weeks of combined training were effective in significantly improving peak oxygen consumption (V ˙O2 peak), maximal workload (Watt max), maximal strength, and flexibility, without adverse cardiac events, cardiac device dysfunction, or ICD shocks.

Hence, despite the presence of cardiac devices, adaptations to exercise training appear to be similar to those documented in the healthy population and other populations of cardiac patients [3,4,5,6,7]. Our findings are clinically relevant because strength training is currently highly recommended, in addition to the aerobic type, in cardiac rehabilitation [26], particularly in elderly patients [26,27].

The benefits of resistance training have been demonstrated on body composition, the cardiovascular system, glucose metabolism, dyslipidemia, quality of life and sarcopenia [28,29,30,31]. Marzolini et al. [32] compared aerobic training alone versus aerobic plus resistance training, demonstrating that combined training was more effective on body composition, strength, and some indicators of cardiovascular fitness. However, most of the studies performed so far refer to patients with ischemic heart disease or heart failure [19,20,32,33,34].

Our patients also underwent flexibility training that resulted in improvements in the anterior-lateral flexibility of the core and in the range of motion of the scapula-humeral joint, in line with the results of Hotta et al. [35] obtained from patients with myocardial infarction.

To our knowledge, this is the first study to address the effectiveness and safety of aerobic, strength and flexibility training in cardiac patients with implanted devices.

These findings may have a meaningful impact on everyday life activities, contributing to the improvement of CRF and quality of life in patients with implantable cardiac devices, particularly in the elderly.

### Limitations of Study

One of the limitations of our study might be represented by the absence of a control group adopting exclusively an aerobic training program. Such a limitation is due to the acknowledgement that a comprehensive training program that includes strength and flexibility would lead to greater health improvements compared to aerobic exercise alone [29,31]. Thus, we decided not to deprive patients of the best health results achievable through a comprehensive training program

Another limitation of the study is the small sample. As such, it should be regarded as a “proof of concept” study. Furthermore, the possible interference of some drugs on the effects induced by the exercise training program cannot be entirely excluded. However, pharmacological therapy did not change throughout the study.

## 5. Conclusions

Our data demonstrate the efficacy of combined exercise training on cardiopulmonary parameters, muscle strength and flexibility in patients with PMs or ICDs, without adverse cardiac events or ICD shocks.

Therefore, eight weeks of combined (aerobic, strength and flexibility) exercise training appears sufficient and safe for improving the functional capacity and quality of life of patients with implantable cardiac devices.

More studies with higher numbers of patients and longer follow-up are needed to confirm these data.

## Figures and Tables

**Table 1:** Clinical and demographic characteristics of patients with implantable cardiac devices.

Clinical and Demographic Characteristics		
**Age**	**N=**	**%**
40–60	4	13
61–80	15	50
81–95	11	37
**CV risk factors**		
Hypertension	9	30
Obesity	5	17
Diabetes Mellitus	4	13
Dyslipidemia	6	20
Smoking	3	10
**ICD or PM indication**		
Atrial fibrillation/atrial flutter	11	37
Complete atrium-ventricular block	6	20
Sick sinus syndrome	6	20
Post-ischemic dilated cardiomyopathy	5	17
Hypertrophic cardiomyopathy	1	3
Recurrent syncope	1	3
**Type of device**		
(PM) dual chamber	17	57
(PM) single chamber	8	27
ICD	5	16
**Medications**		
Diuretics	26	87
Antiarrhytmics	15	50
Angiotensin II receptor blockers	10	33
Betablockers	9	30
Vitamin K antagonists	8	27
Calcium antagonists	8	27
ACE inhibitors	7	23
Statins	6	20
Direct oral anticoagulants	5	17
Aspirin	4	13
Digitalis	4	13
Clopidogrel	2	7
Alfa-blockers	2	7
Nitroglycerin	1	3

**Table 2:** Pre- and post-training data. HR, heart rate; SBP, systolic blood pressure; DBP, diastolic; R, right; L, left.

Aerobic Fitness				Muscle Strength (kg)				Flexibility (cm)			
Parameter	PRE	POST	*p*	Parameter	PRE	POST	*p*	Parameter	PRE	POST	*p*
Basal HR (bpm)	69 ± 6	69 ± 7	0.4	Quadriceps	21 ± 18	29 ± 16	0.000003	Sit and reach	−19 ± 11.7	−15 ± 11.7	0.003
Basal SBP (mmHg)	122 ± 72	121 ± 12	0.3	Biceps brachii	11 ± 5	15 ± 9	0.02	Back scratch R	−19 ± 11.6	−15 ± 10.0	0.02
Basal DBP (mmHg)	74 ± 7	74 ± 11	0.2	Triceps brachii	7 ± 3	9 ± 4	0.003	Back scratch L	−21 ± 17.7	−18 ± 13.5	0.3
Peak power (watt)	87 ± 30.7	108 ± 37.0	0.001	Deltoid	6 ± 2	7 ± 3	0.01	Lateral R	−44 ± 1.4	−43 ± 9.5	0.1
Peak HR (bpm)	96 ± 19.5	106 ± 22.6	0.008					Lateral L	−43 ± 6.0	−45 ± 8.7	0.2
V ˙O2peak (mL/kg/min)	15 ± 3.8	17 ± 4.1	0.001								
V ˙O2peak (mL/min)	1232 ± 386.9	1413 ± 494	0.02								
LT V ˙O2 (mL/kg/min)	10 ± 2.2	11 ± 2.1	0.4								
Peak SBP (mm Hg)	156 ± 19.8	156 ± 23.5	0.4								
Peak DBP (mm Hg)	77 ± 10.7	72 ± 11.1	0.009								
V ˙O2/HR average value	13 ± 3.9	12 ± 5	0.73								

## Data Availability

The data presented in this study are available on request from the corresponding author.

## References

[1] Proclemer A., Zecchin M., D’Onofrio A., Ricci R.P., Boriani G., Rebellato L., Ghidina M., Bianco G., Bernardelli E., Miconi A. (2019). The Pacemaker and Implantable Cardioverter-Defibrillator Registry of the Italian Association of Arrhythmology and Cardiac Pacing—Annual report 2017. G. Ital. Cardiol..

[2] Lavie C.J., Ozemek C., Carbone S., Katzmarzyk P.T., Blair S.N. (2019). Sedentary Behavior, Exercise, and Cardiovascular Health. Circ. Res..

[3] Kaminsky L.A., Arena R., Ellingsen Ø., Harber M.P., Myers J., Ozemek C., Ross R. (2019). Cardiorespiratory fitness and cardiovascular disease—The past, present, and future. Prog. Cardiovasc. Dis..

[4] Pepera G., Bromley P.D., Sandercock G.R.H. (2013). A pilot study to investigate the safety of exercise training and testing in cardiac rehabilitation patients. Br. J. Cardiol..

[5] Aoyama D., Miyazaki S., Hasegawa K., Nagao M., Kakehashi S., Mukai M., Sekihara T., Nodera M., Eguchi T., Aiki T. (2021). Cardiac rehabilitation after catheter ablation of atrial fibrillation in patients with left ventricular dysfunction. Heart Vessels.

[6] Ambrosetti M., Abreu A., Corrà U., Davos C.H., Hansen D., Frederix I., Iliou M.C., Pedretti R.F., Schmid J.-P., Vigorito C. (2020). Secondary prevention through comprehensive cardiovascular rehabilitation: From knowledge to implementation. 2020 update. A position paper from the Secondary Prevention and Rehabilitation Section of the European Association of Preventive Cardiology. Eur. J. Prev. Cardiol..

[7] Hansen D., Abreu A., Ambrosetti M., Cornelissen V., Gevaert A., Kemps H., Laukkanen J.A., Pedretti R., Simonenko M., Wilhelm M. (2021). Exercise intensity assessment and prescription in cardiovascular rehabilitation and beyond: Why and how: A position statement from the Secondary Prevention and Rehabilitation Section of the European Association of Preventive Cardiology. Eur. J. Prev. Cardiol..

[8] Isaksen K., Morken I.M., Munk P.S., Larsen A.I. (2012). Exercise training and cardiac rehabilitation in patients with implantable cardioverter defibrillators: A review of current literature focusing on safety, effects of exercise training, and the psychological impact of programme participation. Eur. J. Prev. Cardiol..

[9] Alswyan A.H., Liberato A.C.S., Dougherty C.M. (2018). A Systematic Review of Exercise Training in Patients with Cardiac Implantable Devices. J. Cardiopulm. Rehabil. Prev..

[10] Iliou M.C., Blanchard J.C., Lamar-Tanguy A., Cristofini P., Ledru F. (2016). Cardiac rehabilitation in patients with pacemakers and implantable cardioverter defibrillators. Monaldi Arch. Chest Dis..

[11] Belardinelli R., Capestro F., Misiani A., Scipione P., Georgiou D. (2006). Moderate exercise training improves functional capacity, quality of life, and endothelium-dependent vasodilation in chronic heart failure patients with implantable cardioverter defibrillators and cardiac resynchronization therapy. Eur. J. Cardiovasc. Prev. Rehabil..

[12] Vanhees L., Schepers D., Heidbüchel H., Defoor J., Fagard R. (2001). Exercise performance and training in patients with implantable cardioverter-defibrillators and coronary heart disease. Am. J. Cardiol..

[13] Allison T.G. (2013). Changing medical culture to promote physical activity in secondary prevention of coronary artery disease. Eur. Heart J..

[14] Binder R.K., Wonisch M., Corra U., Cohen-Solal A., Vanhees L., Saner H., Schmid J.-P. (2008). Methodological approach to the first and second lactate threshold in incremental cardiopulmonary exercise testing. Eur. J. Cardiovasc. Prev. Rehabil..

[15] Bjarnason-Wehrens B., Mayer-Berger W., Meister E., Baum K., Hambrecht R., Gielen S. (2004). German Federation for Cardiovascular Prevention and Rehabilitation. Recommendations for resistance exercise in cardiac rehabilitation. Recommendations of the German Federation for Cardiovascular Prevention and Rehabilitation. Eur. J. Cardiovasc. Prev. Rehabil..

[16] Mazza A., Camera F., Maestri A., Longoni F., Patrignani A., Gualco A., Opasich C., Cobelli F. (2007). Elderly patient-centered rehabilitation after cardiac surgery. Monaldi Arch. Chest Dis..

[17] Steinhaus D.A., Lubitz S.A., Noseworthy P.A., Kramer D.B. (2019). Exercise Interventions in Patients with Implantable Cardioverter-Defibrillators and Cardiac Resynchronization Therapy: A Systematic Review and Meta-Analysis. J. Cardiopulm. Rehabil Prev..

[18] Merritt J.L., McLean T.J., Erickson R.P., Offord K.P. (1986). Measurement of trunk flexibility in normal subjects: Reproducibility of three clinical methods. Mayo Clin. Proc..

[19] Piccini J.P., Hellkamp A.S., Whellan D.J., Ellis S.J., Keteyian S.J., Kraus W.E., Hernandez A.F., Daubert J.P., Piña L., O’Connor C.M. (2013). HF-ACTION Investigators. Exercise training and implantable cardioverter-defibrillator shocks in patients with heart failure: Results from HF-ACTION (Heart Failure and A Controlled Trial Investigating Outcomes of Exercise TraiNing). JACC Heart Fail..

[20] Pandey A., Parashar A., Moore C., Ngo C., Salahuddin U., Bhargava M., Kumbhani D.J., Piccini J.P., Fonarow G.C., Berry J.D. (2017). Safety and Efficacy of Exercise Training in Patients with an Implantable Cardioverter-Defibrillator: A Meta-Analysis. JACC Clin. Electrophysiol..

[21] Sandercock G.R., Cardoso F., Almodhy M., Pepera G. (2013). Cardiorespiratory fitness changes in patients receiving comprehensive outpatient cardiac rehabilitation in the UK: A multicentre study. Heart.

[22] Pryzbek M., MacDonald M., Stratford P., Richardson J., McQuarrie A., McKelvie R., Tang A. (2021). Long-Term Enrollment in Cardiac Rehabilitation Benefits of Cardiorespiratory Fitness and Skeletal Muscle Strength in Females with Cardiovascular Disease. Womens Health Rep..

[23] Stefanakis M., Batalik L., Papathanasiou J., Dipla L., Antoniou V., Pepera G. (2021). Exercise-based cardiac rehabilitation programs in the era of COVID-19: A critical review. Rev. Cardiovasc. Med..

[24] Perrone M.A., Donatucci B., Salvati A., Gualtieri P., De Lorenzo A., Romeo F., Bernardini S. (2019). Inflammation, oxidative stress and gene expression: The postprandial approach in professional soccer players to reduce the risk of muscle injuries and early atherosclerosis. Med. Sport.

[25] Tutor A., Lavie C.J., Kachur S., Dinshaw H., Milani R.V. (2022). Impact of cardiorespiratory fitness on outcomes in cardiac rehabilitation. Prog. Cardiovasc. Dis..

[26] Guidelines for Exercise Testing and Prescription, 11th edition 2021. https://www.acsm.org/read-research/books.

[27] Khadanga S., Savage P.D., Ades P.A. (2019). Resistance Training for Older Adults in Cardiac Rehabilitation. Clin. Geriatr. Med..

[28] Lin X., Zhang X., Guo J., Roberts C.K., McKenzie S., Wu W.C., Liu S., Song Y. (2015). Effects of Exercise Training on Cardiorespiratory Fitness and Biomarkers of Cardiometabolic Health: A Systematic Review and Meta-Analysis of Randomized Controlled Trials. J. Am. Heart Assoc..

[29] Williams M.A., Haskell W.L., Ades P.A., Amsterdam E.A., Bittner V., Franklin B.A., Gulanick M., Laing S.T., Stewart K.J., American Heart Association Council on Clinical Cardiology (2007). Resistance exercise in individuals with and without cardiovascular disease: 2007 update: A scientific statement from the American Heart Association Council on Clinical Cardiology and Council on Nutrition, Physical Activity, and Metabolism. Circulation.

[30] Perrone M.A., Feola A., Pieri M., Donatucci B., Salimei C., Lombardo M., Perrone A., Parisi A. (2021). The Effects of Reduced Physical Activity on the Lipid Profile in Patients with High Cardiovascular Risk during COVID-19 Lockdown. Int. J. Environ. Res. Public Health.

[31] Fealy C.E., Nieuwoudt S., Foucher J.A., Scelsi A.R., Malin S.K., Pagadala M., Cruz L.A., Li M., Rocco M., Burguera B. (2018). Functional high-intensity exercise training ameliorates insulin resistance and cardiometabolic risk factors in type 2 diabetes. Exp. Physiol..

[32] Marzolini S., Oh P., Brooks D. (2012). Effect of combined aerobic and resistance training versus aerobic training alone in individuals with coronary artery disease: A meta-analysis. Eur. J. Prev. Cardiol..

[33] Caminiti G., Iellamo F., Perrone M.A., D’Antoni V., Catena M., Manzi V., Morsella V., Franchini A., Volterrani M. (2021). Central Hemodynamic Adjustments during Post-Exercise Hypotension in Hypertensive Patients with Ischemic Heart Disease: Concurrent Circuit Exercise versus High-Intensity Interval Exercise. A Preliminary Study. J. Clin. Med..

[34] Caminiti G., Perrone M.A., Iellamo F., D’Antoni V., Catena M., Franchini A., Volterrani M. (2022). Acute Left Atrial Response to Different Eccentric Resistance Exercise Loads in Patients with Heart Failure with Middle Range Ejection Fraction: A Pilot Study. J. Pers. Med..

[35] Hotta K., Kamiya K., Shimizu R., Yokoyama M., Nakamura-Ogura M., Tabata M., Kamekawa D., Akiyama A., Kato M., Noda C. (2013). Stretching exercises enhance vascular endothelial function and improve peripheral circulation in patients with acute myocardial infarction. Int. Heart J..

